# Bifidobacteria define gut microbiome profiles of golden lion tamarin (*Leontopithecus rosalia*) and marmoset (*Callithrix* sp.) metagenomic shotgun pools

**DOI:** 10.1038/s41598-023-42059-4

**Published:** 2023-09-21

**Authors:** Joanna Malukiewicz, Mirela D’arc, Cecilia A. Dias, Reed A. Cartwright, Adriana D. Grativol, Silvia Bahadian Moreira, Antonizete R. Souza, Maria Clotilde Henriques Tavares, Alcides Pissinatti, Carlos R. Ruiz-Miranda, André F. A. Santos

**Affiliations:** 1 Primate Genetics Laboratory, German Primate Center, Leibniz Institute for Primate Research, Göttingen, 37077 Germany; 2 Instituto de Medicina Tropical de São Paulo, Universidade de São Paulo, São Paulo, SP 05403-000 Brazil; 3 Laboratório de Diversidade e Doenças Virais, Departamento de Genética, Universidade Federal do Rio de Janeiro, Rio de Janeiro, RJ Brazil; 4 Centro de Primatologia, Universidade de Brasília, Brasília, Brazil; 5 School of Life Sciences and the Biodesign Institute, Arizona State University, Tempe, AZ 85281 USA; 6 Sana Kombucha ME, Macaé, RJ 27910-060 Brazil; 7 Centro de Primatologia do Rio de Janeiro, Instituto Estadual do Ambiente, Rio de Janeiro, Brazil; 8 Laboratorio das Ciencias Ambientais, Centro de Biociencias e Biotecnologia, Universidade Estadual do Norte Fluminense, Campos dos Goytacazes, RJ 28013-602 Brazil

**Keywords:** Microbiome, Zoology

## Abstract

Gut microbiome disruptions may lead to adverse effects on wildlife fitness and viability, thus maintaining host microbiota biodiversity needs to become an integral part of wildlife conservation. The highly-endangered callitrichid golden lion tamarin (GLT-*Leontopithecus rosalia*) is a rare conservation success, but allochthonous callitrichid marmosets (*Callithrix*) serve as principle ecological GLT threats. However, incorporation of microbiome approaches to GLT conservation is impeded by limited gut microbiome studies of Brazilian primates. Here, we carried out analysis of gut metagenomic pools from 114 individuals of wild and captive GLTs and marmosets. More specifically, we analyzed the bacterial component of ultra filtered samples originally collected as part of a virome profiling study. The major findings of this study are consistent with previous studies in showing that *Bifidobacterium*, a bacterial species important for the metabolism of tree gums consumed by callitrichids, is an important component of the callitrichid gut microbiome - although GTLs and marmosets were enriched for different species of *Bifidobacterium*. Additionally, the composition of GLT and marmoset gut microbiota is sensitive to host environmental factors. Overall, our data expand baseline gut microbiome data for callitrichids to allow for the development of new tools to improve their management and conservation.

## Introduction

The gut microbiome provides the host with a number of essential metabolic, immune, and physiological functions^1,2^, and various factors influence gut microbiome community structure. Host phylogeny shapes gut microbiome composition through vertical transmission of microbiota and host-microbe interactions related to immune genes^3,4^. Diet, on the other hand, individualizes the gut microbiome profiles of a given host by enriching the gut with microbiota related to a host’s feeding strategy^5,6^. In wild mammals, the microbiome functional landscape is significantly associated with host diet, life span, body mass, and social structure^7^. Importantly, the physiological and functional benefits of host-associated microbial communities are susceptible to disruptions by several anthropogenic factors like deforestation, land-use change, urbanization, and captivity^8–10^.

As gut microbiome disruptions may lead to adverse effects on wildlife fitness and viability, there is a growing awareness that maintaining the biodiversity of host-associated microbiota may need to become an integral part of wildlife management and conservation practices^2,9^. The recovery of the highly endangered golden-lion tamarin (*Leontopithecus rosalia*, GLT) within its native Brazilian Atlantic Forest biome, one of the world’s 25 biodiversity hot spots^11^, is a rare conservation success story^12^. Although conservation of the species continues through strategic plans coordinated by the Brazilian organization *Associação Mico-Leão-Dourado *(AMLD)^13^, the species remains threaten by anthropogenic activities related to land use and urbanization that lead to habitat degradation and fragmentation^14,15^. Furthermore, the presence of anthropogenic marmoset hybrids, which arose after human introductions of allochthonous marmoset species (common marmosets *Callithrix jacchus* and black-tufted marmosets *C. penicillata*) into forest fragments located within the native GLT range^16,17^, represent additional ecological and health threats to GLT recovery^18^. Incorporation of microbiome approaches for future GLT conservation efforts is currently impeded by a restricted understanding of the gut microbiome of most Brazilian primates. With the exception of a number 16S rRNA and ITS microbial profiling studies (e.g.^19–22^), microbiome studies of Brazilian primates are limited.

Prior microbiome work has shown that the Callitrichidae family, which includes GLTs and marmosets, possesses the highest average primate gut microbiome abundance of *Bifidobacterium* (> 30%) as well as significant host co-evolutionary signal with *Bifidobacterium*^21^. This bacterial genus is a natural commensal that functionally supports carbohydrate metabolism within their hosts^21,23^ and is involved in other crucial host biological processes (see^24^). GLTs and marmosets both exploit viscous plant exudates composed of polysaccharides as part of their dietary intake, albeit to a different degree. A number of marmoset species are considered obligate exudivores^25^, and some species exhibit high morphological specialization for exudivory^26,27^. In wild common marmosets, exudate consumption provides key nutrients (calcium, protein) for balancing their diet, especially during the dry season. Exudates vary in complex carbohydrate content (38–81%) and can contain more calcium than insects, more protein than other plant sources, and even high water content^28^. As frugivore-insectivores, GLTs eat exudates opportunistically^29,30^, but exudates overall are considered an essential part of the callitrichid diet^31^. In fact, *Bifidobacterium* seems to play a key functional role in the wild marmoset gut microbiome^19^, and captivity reduces gut *Bifidobacterium* abundance in marmosets as well as other primate hosts^19,21^. It is indeed thought that such changes in host gut microbiome composition between the wild and captivity affect the health and viability of captive marmosets^19^.

To further expand understanding of the callitrichid gut microbiome, especially that of GLTs, we took advantage of opportunistically-available data from ultra-filtered metagenomic sequencing short-read pooled libraries (metagenomic pools hereafter) from feces and anal swabs of captive and wild GLTs and marmosets. The biological samples were originally collected as part of a virome profiling study, and were therefore enriched for viral-like particles (VPLs) through ultra-filtration and nuclease treatment. Nonetheless, the presence of non-viral genetic material is common in such genomic libraries regardless of enrichment protocol^32,33^. Although the non-viral genetic material is usually considered a contaminant in virome studies^33^, from the microbiome perspective, such material may still contain valuable taxonomic and functional bacterial information.

Here, we carried out metagenomic analysis of callitrichid VPL pools using both microbial community and metagenome-assembled genome (MAG) approaches from a total of 114 individuals of wild and captive GLTs, captive black-tufted marmosets (*C. penicillata*) and wild *C. jacchus* x *C. penicillata* hybrids. We hypothesize that the microbiome component of VPL-enriched genomic libraries represents only the most abundant bacterial taxa present within the sampled host material. Further, we hypothesize that host gut microbiome metagenomic pools from both GLTs and marmosets show strong enrichment for *Bifidobacterium*, but that this bacterial taxon is more abundant in the gut of wild callitrichids than that of captive callitrichids. As it has been previously shown that *Bifidobacterium* species are specific for different host species and taxa^21^, we predict that the gut microbiome profiles of *Leontopithecus* and *Callithrix* metagenomic pools will respectively possess distinct *Bifidobacterium* species. We do expect the functional profile of the bacterial component of our analyzed host gut microbiome metagenomic pools to be biased towards carbohydrate function in both GLTs and marmosets.

## Methods

### Sample collection

**Figure 1:**
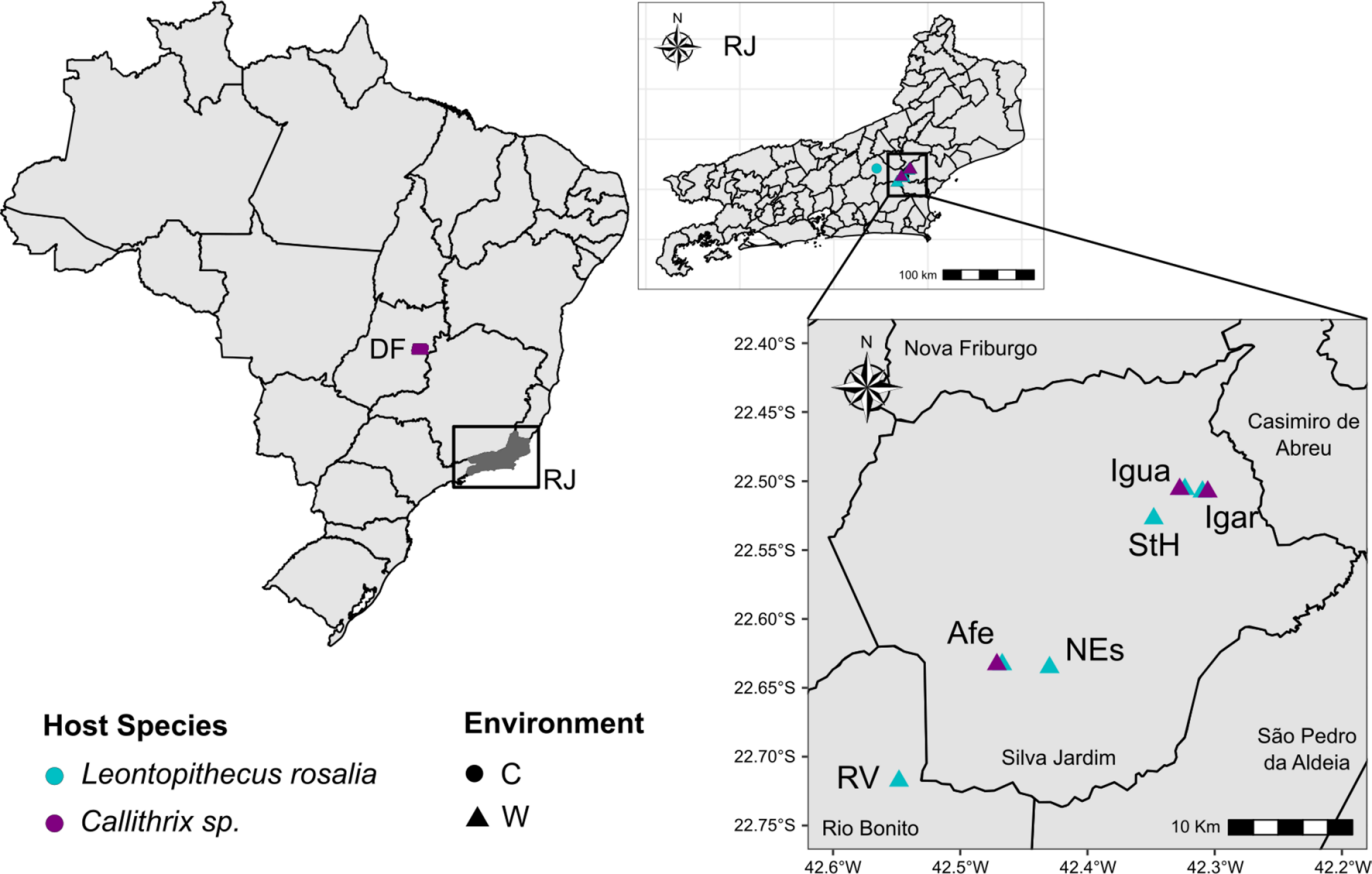
Map showing the sample collection sites with host environment (C/circle = captive and W/triangle = wild) and host species information (*Leontopithecus rosalia* shown as teal and *Callithrix* sp. shown as purple). The map inset represents the sampling localities of Silva Jardim and Rio Bonito, Rio de Janeiro state (RJ), Brazil. The *Callithrix* sampling locality of Brasília, Federal District is also shown on the main map as “DF.”

We studied six readily accessible groups of wild golden lion tamarins (*Leontopithecus rosalia*; GLT; n = 71) and four groups of wild marmosets (*Callithrix* sp.; n = 9) that were habituated to regular human contact and consistently monitored by AMLD in Silva Jardim and Rio Bonito municipalities in Rio de Janeiro state, Brazil (Fig. 1). Previous genetic analysis of marmoset populations from this region indicate that these populations comprise an anthropogenic hybrid swarm descended from introduced populations of *C. jacchus* and *C. penicillata*^17,34^. Callitrichid groups were sampled in several small fragments of the Atlantic Forest at the São João river basin: Afetiva (Afe; GLT = 12 and marmoset = 4), Igarapé (Igar; GLT = 11 and marmoset = 2), Iguape (Igua; GLT = 5 and marmoset = 3), Nova Esperança (NEs; GLT = 16), Santa Helena 1 (StH; GLT = 13) and Rio Vermelho (RV; GLT = 14). Wild marmosets and tamarins were immobilized for routine veterinary check-ups with injection of ketamine (approximately 10–15 mg/kg) into the intramuscular region of the inner thigh. Then, fecal samples were collected in 15 mL tubes and this volume was mixed in a proportion of 1:1 with RNA*later*, followed by vigorous homogenization. Tubes were kept at ambient temperature in the field and sent to the Laboratory of Viral Diversity and Disease (LDDV), in the Department of Genetics of the Federal University of Rio de Janeiro (UFRJ), Rio de Janeiro, Brazil, to freeze at − 80 $^\circ$C until processing. Field information collected for each sampled animal included all the following: ID number, group, specie, age, sex, weight and clinical conditions.

We also included in the study a captive GLT group (n = 3) housed at *Centro de Primatologia do Rio de Janeiro* (CPRJ; Primate Center of Rio de Janeiro) located in Guapimirim, Rio de Janeiro, Brazil. Finally, we sampled six marmosets groups of *C. penicillata* (n = 39; 4–6 animals by group) housed at *Centro de Primatologia da Universiade de Brasília* (CP/UnB; Primate Center of the Brasilia University), Brasília, Federal District, Brazil. Animals were kept in enclosures surrounded by natural vegetation and were maintained in couples or in groups according to the rules of IBAMA (Brazilian Institute of Environment and Renewable Natural Resources). The marmosets from CP/UnB were anesthetized also for routine veterinary check-ups and anal swab samples were collected using sterile cotton swabs with plastic shafts. Swab samples were placed in 1.5 mL centrifuge tubes with 500 $\upmu$L of PBS and later excised close to the cotton tip using flame-sterilized scissors. Tubes were kept at − 20 $^\circ$C until processing. For the GLTs from CPRJ, only fecal samples were collected using a similar method as specified for wild GLTs. General information was collect for all sampled animals as: ID number, enclosure, specie, age, sex, weight and clinical conditions. Although our sampling included both anal swabs and fecal samples, several previous studies indicate that anal swabs are reliable proxies for fecal samples^35–38^, thus we treated both sample types as equivalent.

General sampling information is summarized in Fig. 1 and Table 1. Host taxon identification at the genus level, *Callithrix* and *Leontopithecus*, followed previously published phenotype descriptions^34^ and official registered identification by primate captive facilities and the AMLD. Hosts were also classified by their environment as wild (captured as free-range individuals) or captive (maintained in captivity). All samples were collected following the national guidelines and provisions of CONCEA (National Council for Animal Experimentation Control, Brazil), which included animal welfare standard operating procedures. This project was approved by the Ethics Committee on the Use of Animals (CEUA) of UFRJ (reference number 037/14). This study is reported in accordance to ARRIVE guidelines (https://arriveguidelines.org/resources/questionnaire). All methods were carried out in accordance with relevant international guidelines and regulations.

**Table 1:** Information summary on golden lion tamarin (GLT) and marmoset gut metagenomic pools with host taxon, sampling location, sampling coordinates, number of individual hosts included in each pool, and host environment. For sampling locations, the following abbreviations are used: CP/UnB = Centro de Primatologia de Brasília / University of Brasília. For host environment, the following abbreviations are used: W = Wild, C = Captive. For host location the following abbreviations are used: RJ = Rio de Janeiro, DF = Federal District.

Host species	Host common name	Location (ID)	Pool ID	Sample approximate collection geographic coordinates	Sample type	Pool N	Host environment
*Leontopithecus rosalia*	GLT	Afetiva (Afe), Silva Jardim, RJ	MLDAfe	22$^\circ$ 37$'$58.84$''$ S–42$^\circ$ 28$'$1.47$''$ W	Feces	4	W
*Leontopithecus rosalia*	GLT	Afetiva (Afe), Silva Jardim, RJ	LrAfef	22$^\circ$ 37$'$58.84$''$ S–42$^\circ$ 28$'$1.47$''$ W	Feces	8	W
*Leontopithecus rosalia*	GLT	Igarapé (Igar), Silva Jardim, RJ	MLDIgar	22$^\circ$ 30$'$27.41$''$ S–42$^\circ$ 18$'$34.66$''$ W	Feces	3	W
*Leontopithecus rosalia*	GLT	Igarapé (Igar), Silva Jardim, RJ	LrIgarf	22$^\circ$ 30$'$27.41$''$ S–42$^\circ$ 18$'$34.66$''$ W	Feces	8	W
*Leontopithecus rosalia*	GLT	Iguape (Igua), Silva Jardim, RJ	MLDIgua	22$^\circ$ 30$'$20.74$''$ S–42$^\circ$ 19$'$24.27$''$ W	Feces	5	W
*Leontopithecus rosalia*	GLT	Rio Vermelho (RV), Rio Bonito, RJ	MLDRV	22$^\circ$ 43$'$3.26$''$ S–42$^\circ$ 32$'$53.37$''$ W	Feces	9	W
*Leontopithecus rosalia*	GLT	Rio Vermelho (RV), Rio Bonito, RJ	LrRVf	22$^\circ$ 43$'$3.26$''$ S–42$^\circ$ 32$'$53.37$''$ W	Feces	5	W
*Leontopithecus rosalia*	GLT	Nova Esperança (NEs), Silva Jardim, RJ	LrNEs1f	22$^\circ$ 38$'$6.54$''$ S–42$^\circ$ 25$'$47.38$''$ W	Feces	8	W
*Leontopithecus rosalia*	GLT	Nova Esperança (NEs), Silva Jardim, RJ	LrNEs2f	22$^\circ$ 38$'$6.54$''$ S–42$^\circ$ 25$'$47.38$''$ W	Feces	8	W
*Leontopithecus rosalia*	GLT	Santa Helena (StH) 1, Silva Jardim, RJ	LrStH1f	22$^\circ$ 31$'$37.33$''$ S–42$^\circ$ 20$'$52.51$''$ W	Feces	7	W
*Leontopithecus rosalia*	GLT	Santa Helena (StH) 1, Silva Jardim, RJ	LrStH2f	22$^\circ$ 31$'$37.33$''$ S–42$^\circ$ 20$'$52.51$''$ W	Feces	6	W
*Leontopithecus rosalia*	GLT	CPRJ, Guapimirim, RJ	PoolRosalia	22$^\circ$ 29$'$17.458$''$ S–42$^\circ$ 54$'$48.432$''$ W	Feces	3	C
*Callithrix* sp.	Marmoset	Afetiva (Afe), Silva Jardim, RJ	SAfe	22$^\circ$ 37$'$58.84$''$ S–42$^\circ$ 28$'$1.47$''$ W	Feces	4	W
*Callithrix* sp.	Marmoset	Igarapé (Igar), Silva Jardim, RJ	SIgar	22$^\circ$ 30$'$27.41$''$ S–42$^\circ$ 18$'$34.66$''$ W	Feces	2	W
*Callithrix* sp.	Marmoset	Iguape (Igua), Silva Jardim, RJ	SIgua	22$^\circ$ 30$'$20.74$''$ S–42$^\circ$ 19$'$24.27$''$ W	Feces	3	W
*Callithrix* sp.	Marmoset	CP/UnB, DF	P1	15$^\circ$ 56$'$ 54.624$''$ S–47$^\circ$ 56$'$ 2.575$''$ W	Anal Swab	5	C
*Callithrix* sp.	Marmoset	CP/UnB, DF	P2	15$^\circ$ 56$'$ 54.624$''$ S–47$^\circ$ 56$'$2.575$''$ W	Anal Swab	5	C
*Callithrix* sp.	Marmoset	CP/UnB, DF	P3	15$^\circ$ 56$'$54.624$''$ S–47$^\circ$ 56$'$ 2.575$''$ W	Anal Swab	5	C
*Callithrix* sp.	Marmoset	CP/UnB, DF	P4	15$^\circ$ 56$'$ 54.624$''$ S–47$^\circ$56$'$ 2.575$''$ W	Anal Swab	4	C
*Callithrix* sp.	Marmoset	CP/UnB, DF	P7	15$^\circ$ 56$'$ 54.624$''$ S–47$^\circ$ 56$'$ 2.575$''$ W	Anal Swab	6	C
*Callithrix* sp.	Marmoset	CP/UnB, DF	P8	15$^\circ$ 56$'$ 54.624$''$ S–47$^\circ$ 56$'$ 2.575$''$ W	Anal Swab	6	C

### Sample processing and sequencing

The molecular protocol was conducted as follows: after thawing and vigorous homogenization for 1 min, 1 mL of fecal sample was disrupted by Lysing Matrix E extraction beads (MPbio) and clarified by centrifugation at 6000*g* for 10 min at 4 $^\circ$C. Between 100–250 $\upmu$L of fecal supernatants or PBS direct from swab samples was mixed with samples from animals of the same location, resulting in 18 pools (GLT = 9 and Marmoset = 9). Sampling pools are summarized in Table 1. The pooled samples were filtered through an Ultra-free-MC HV 0.45 $\upmu \hbox {m}$ sterile filter (Millipore, UFC30HV0S). The filtrates were submitted to sucrose density gradient ultracentrifuge separation at 35,000*g* for 90 min at 4 $^\circ$C and degradation of unprotected nucleic acid by nuclease digestion at 37 $^\circ$C for 60 min. Remaining nucleic acid (DNA and RNA) were then isolated using QIAamp^®^ MinElute^®^ Virus Spin kit (Qiagen), followed the manufacturer’s instructions, with the following modifications: (i) Carrier RNA was omitted from the AL Buffer; (ii) the protease was resuspended in AVE Buffer, instead of Protease Resuspension Buffer; (iii) the washing step with AW1 was suppressed; (iv) the final elution was performed in 20 $\upmu \hbox {L}$ of ultra-pure water. Thereafter, a RT-PCR reaction was performed with the SuperScript^®^ III First-Strand Synthesis System (Invitrogen) for first-strand cDNA synthesis from RNA, using random primers, while preserving DNA. The second-strand cDNA synthesis was performed using a DNA Polymerase I Large (Klenow 3$'$–5$'$ exo) Fragment (New England Biolabs^®^). All those reactions were conducted according to the manufacturer’s instructions. Total DNA (DNA and cDNA) quantification was performed using the High Sensitivity dsDNA Assay kit in a Qubit 2.0 Fluorometer (Thermo Fisher Scientific). The libraries were constructed using the Nextera XT - DNA Library Preparation Kit (Illumina), purified with the Agencourt AMPure XP -PCR Purification (Beckman Coulter) kit and quantified using both High Sensitivity DNA Kits from Qubit 2.0 Fluorometer (Thermo Fisher Scientific) and 2100 Bioanalyzer (Agilent Technologies). The sequencing was conducted by applying 2 pM of each library in the MiSeq Illumina platform using the MiSeq V2 300-cycle kit (Illumina) in paired-end mode 2 $\times$ 150 bp with dual barcode for each pooled sample.

### Bioinformatics and statistical analysis

#### Metagenomic community profiling

Initially, analyses for quality filtering, taxonomic profiling, and functional profiling of callitrichid gut microbiomes was carried out with the bioBakery metagomic environment^39^. We first used KneadData v0.7.10 (https://huttenhower.sph.harvard.edu/kneaddata/) with default settings for quality control of raw pair-ended fastq files from individual pooled metagenomic sequencing libraries, which included removal of potential host reads and trimming of low quality regions from the reads. Kneaddata used Trimmomatic v0.39^40^ for trimming and Bowtie2 v 2.4.2^41^ for removal of contaminating host reads. For reference host genomes, we combined four publicly available Neotropical primate genomes from GenBank (*Saimiri boliviensis boliviensis* - GCA_000235385.1; *Callithrix jacchus* - GCA_002754865.1; *Aotus nancymaae* - GCA_000952055.2; *Cebus capucinus imitator* - GCA_001604975.1) into a single fasta file, which was then turned into a reference database for use by Bowtie2 and thus promoted a deep cleaning of the host reads, especially in the case of the GLT that does not have a published genome. We installed the CHOCOPhlAn_201901 database for use with MetaPhlAn v3.0.7. Then we profiled the composition of microbial community composition of our pooled metagenomic shotgun sequencing data with MetaPhlAn using the following command “metaphlan *.kneaddata_paired_1.fastq, *.kneaddata_paired_2.fastq –bowtie2out *.bowtie2.counts.bz2 –input_type fastq -o *.profiled_metagenome.counts.txt -t rel_ab_w_read_stats.” The “-t rel_ab_w_read_stats” option was used to profile each pooled metagenome in terms of relative abundances and estimate the number of reads for each identified bacterial clade. Resulting MetaPhlAn count tables of bacterial species from individual metagenomic pools were merged into one large table (Supplementary Table S1) with R v4.2.2^42^. However, at this point, metagenomic pools LrRVf, LrIgarf, LrNEs1f, and MLDRV were excluded as they did not contain sequencing reads identified as bacteria during the MetaPhlAn analysis. Code for the above analyses is available at https://github.com/Callithrix-omics/callitrichidae_microbiome/blob/main/Metagenomic_Community_Profiling/biobakery.sh.

The merged MetaPhlAn table was then read into R for alpha and beta diversity analysis with phyloseq 1.34.0^43^ and vegan 2.5-7^44^. Input files to create the phyloseq object are accessible at https://github.com/Callithrix-omics/callitrichidae_microbiome/blob/main/additional_files/phyloseq_OTU.tsv and https://github.com/Callithrix-omics/callitrichidae_microbiome/blob/main/additional_files/phyloseq_taxaonomy.tsv. We first normalized bacterial read counts in each gut metagenomic pool using median bacterial abundance values across the entire data set. The resulting normalized counts by host taxon are listed in Supplementary Table S2 and by host environment in Supplementary Table S3. We first created a phyloseq object which was then turned into a vegan compatible data object. We calculated the Shannon diversity index with vegan to measure the gut microbiome alpha diversity of callitrichid metagenomic pools while classifying hosts by genus and environment, respectively. To better understand the effects of both host environment and host genus on callitrichid gut microbiome alpha diversity, we fitted a two-way ANOVA model. Shannon index measures were used as model response variables, and host environment and host genus as independent variables. Levene’s test indicated homogeneous variances in the two independent variables (F(3,14) = 1.10, p = 0.38). No interaction between the independent variables was used, as we assumed host genus was independent of host environment. A diagnostic residuals Q-Q plot was used to check the data for normality. The code for the analyses described in this paragraph is available specifically at https://github.com/Callithrix-omics/callitrichidae_microbiome/blob/main/Metagenomic_Community_Profiling/R_MLD_marmoset_microbiome_vegan_phyloseq.rmd.

Phyloseq was also used to estimate gut microbiome bacterial species abundance for callitrichid metagenomic pools and construct a bacterial abundance plot. To test for significance in differential bacterial taxa abundance according to host environment and host genus, respectively, we used LEfSe^45^ at species level for bacterial taxa. The merged table of MetaPhlAn bacterial species counts within each pooled sample library, was loaded into the LEfSe submodule within MicrobiomeAnalyst^46^. The analysis was carried out with the default settings of a FDR-adjusted p-value cutoff set to 0.1 and the log LDA cut-off at 2.0. Prior to carrying out the LEfSe analysis, data were normalized in MicrobiomeAnalyst with settings of the ‘Low count filter’ set to ‘Mean abundance value’ and the option under ‘Low variance filter’ set to 10% based on the interquantile range. Next, at the data normalization step, data were scaled by ‘total sum scaling,’ and we did not apply any data transformations.

To explore beta diversity of the callitrichid gut microbiome in R, using the same phyloseq object as above, we calculated the Bray–Curtis dissimilarity indices for each host, and then used the indices to make a Principle Coordinates Analysis (PCoA) plot in vegan. We superimposed both host environmental and host taxon information onto the PCoA plot. To understand the effects that host environment and host taxon had on marmoset gut microbiome Bray–Curtis dissimilarity indices, we used adonis2 function in the phyloseq package^43^. We fitted PERMANOVA^47^ models which included the marginal effects of host environment and host taxon as independent variables and Bray–Curtis dissimilarity indices as the dependent variables. The PERMANOVA models were run with the adnois2 function. PERMANOVA post-hoc tests of Bray–Curtis dissimilarity indices were carried out as pairwise adonis tests with the adonis.pair function from the the EcolUtils^48^ R package. The test was run for 1000 permutations and p-values were corrected by the false discovery rate. The code for the analyses described in this paragraph is available at https://github.com/Callithrix-omics/callitrichidae_microbiome/blob/main/Metagenomic_Community_Profiling/R_MLD_marmoset_microbiome_vegan_phyloseq.rmd.

#### Metagenomic community functional profiling

In order to identify functional gut microbiome pathways among our pooled samples we used HUMAnN3 v3.0.0.alpha.4^49^. Since HUMAnN does not utilize pair-ended fastqs, we first merged all such pairs of Kneaddata-filtered fastqs into a single file that served as input to HUMAnN. We ran the program under default settings with the ChocoPhlAn pangenome database (part of the bioBakery environment) to profile callitrichid functional gut microbiome Metacyc^50^ metabolic pathway abundance. Analyses ran for individual sample pools were merged with the HUMAnN humann_renorm_tabl command and normalized from reads per kilobase (RPK) units to counts per million (CPM). After filtering out unidentified functional pathways among sample metagenomic pools, we conducted a multivariate analysis to search for significant differential abundance between gut microbiome function pathways among our sample pools and host genus and environment with MaAsLin 2.0^51^. Names of resulting HUMAnN3 pathways and recoded numbering of these pathways is available at https://github.com/Callithrix-omics/callitrichidae_microbiome/blob/main/additional_files/bacterioma_pathways_coded_Humann_Maasalin.tsv. The input for MaAsLin 2.0 is provide at https://github.com/Callithrix-omics/callitrichidae_microbiome/blob/main/additional_files/bacterioma_pathways.cleaned.transpose.dummy.tsv. We ran this program in R by fitting a fixed effects model (“expr  Host Genus + Host Environment”). For functional pathways with differential abundance in the callitrichid gut, we identified their higher order ‘superclass’ in the Metacyc database (https://metacyc.org). Finally, for significantly differentially abundant gut microbiome functional pathways in our sample pools, we calculated contributions of bacterial species to each pathway with the Humann3 humann_barplot command. Each given pathway was considered as the focal feature within the humann_barplot command. Code for the HUMAnN analyses described above is available at https://github.com/Callithrix-omics/callitrichidae_microbiome/blob/main/Metagenomic_Community_Profiling/biobakery.sh. Code for the MaAsLin analysis is provided at https://github.com/Callithrix-omics/callitrichidae_microbiome/blob/main/Metagenomic_Functional_Profiling/R_MLD_marmoset_microbiome_maasalin.rmd.

#### Recovery and characterization of MAGs

All steps of MAG recovery were performed through a co-assembly approach with the KBase platform^52^, using the the same set of raw pair-ended fastq files as used above with the bioBakery metagomic environment. Gut metagenomic pools LrRVf, LrIgarf, LrNEs1f, and MLDRV were excluded from MAG analyses, as they did not contain sequencing reads identified as bacteria during community profiling analysis. Prior to uploading these reads into a Kbase narrative, they were pre-processed for quality in fastp 0.23.4^53^ with default settings for removing low quality reads, removing adaptors, trimming of low quality base calls, and read de-duplication. For co-assembly, we then merged all fastqs together from wild *L. rosalia* sample pools, captive *Callithrix* sample pools, and wild *Callithrix* sample pools, respectively. Only reads from the single sample pool from captive *L. rosalia* were not merged with any additional samples. Merged filtered paired-end reads were uploaded using the KBase app Upload File to Staging from Web v1.0.12 and Import FASTQ/SRA File as Reads from Staging Area^52^, respectively. Bins from each merged set of sample pools were recovered by first assembling metagenome contigs using metaSPades v.3.15.3^54^ and MEGAHIT v1.2.9^35^. As ultimately full MAG analysis was carried out only for data from captive marmoset hosts (see below), we continued with analysis of metaSPADEs contigs as they provided better results than MEGAHIT for captive marmoset gut microbiome pools in terms of N50 and the longest length contig (Supplementary Fig. S1 and Supplementary Fig. S2). We then conducted binning of contigs with CONCOCT v1.1^55^ and MAXBIN2 v2.2.4^56^. Resulting bins from CONCOCT and MAXBIN2 from each co-assembly were optimized using the app DAS Tool v1.1.2^57^ and optimized bins were quality-checked using CheckM v1.0.18. After CheckM bin quality checks, only the captive *Callithrix* optimized co-assembly bins showed completeness above 30% and marker lineage beyond “root.” Thus, from this point we only focused on the 4 recovered captive *Callithrix* optimized co-assembly bins. Although only a single bin (bin.002) out of these four met the minimum Metagenome-Assembled Genome (MIMAG) standards of completeness (> 90%) and contamination (< 5%) for high-quality drafts of MAGS^52^, we included all four due to their relevance for exploratory aims of this study. The four bins were functionally annotated by the Annotate and Distill Assemblies app with DRAM v.0.1.2 (Distilled and Refined Annotation of Metabolism)^58^. Taxonomic classification of MAGs was done with the GTDB-Tk29 v1.76.0^59^ taxonomic classification tool.

### Ethics

Tissues were collected under the approval of the Brazilian Environmental Ministry (SISBIO protocols 17409 and 35931). Biological tissue sampling complied with all institutional, national, and international guidelines.

## Results

### Community profiling of callitrichid metagenomic pools

#### *L.rosalia* and *Callithrix* sp. metagenomic pool gut microbiome alpha and beta-diversity

**Figure 2:**
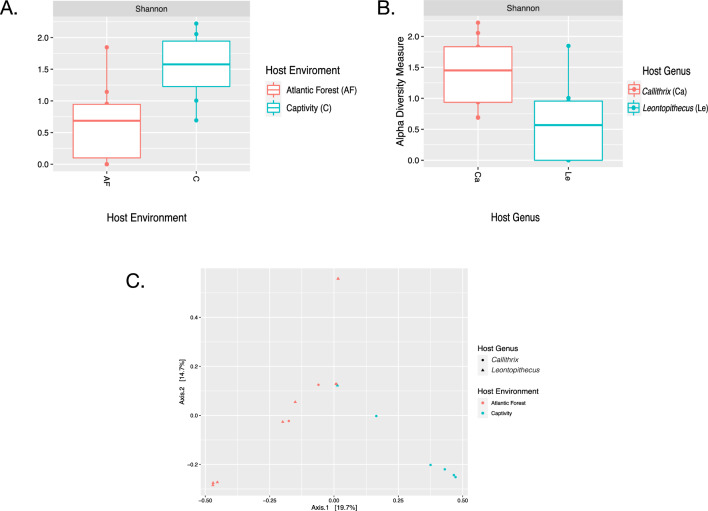
Boxplots of callitrichid gut microbiome Shannon indices for sample pools by host taxon (**A**) and host environment (**B**). Panel (**C**) shows a PCoA plot for host pool gut microbiome beta-diversity measured by the Bray–Curtis dissimilarity index.

Shannon index box plots of callitrichid gut metagenomic pool alpha diversity are shown in Fig. 2. Considering host environment, alpha diversity values were higher in wild hosts than in captive hosts (Fig. 2A). For host genus, gut microbiome alpha diversity was higher for *Callithrix* hosts than for *Leontopithecus* hosts (Fig. 2B). A two-way ANOVA model (Shannon Index $^\sim$ Genus+Environment) was fit to test for differences in host gut microbiome Shannon diversity indices when considering both host environment and host genus. Callitrichid gut microbiome alpha diversity differences due to host genus were significant (ANOVA, F(1) = 8.861, p-value = 0.01), but those due to host environment were not (ANOVA, F(1) = 3.6, p-value = 0.08).

A PCoA plot of host metagenomic pool Bray–Curtis dissimilarity index measures with superimposition of host environment and host taxon is shown in Fig. 2C. This plot shows gut microbiome dissimilarity in the callitrichid gut microbiome when considering host genus as well as host environment. We fit a PERMANOVA model with independent variable margins, to explain the effects of host environment and host taxon on callitrichid gut microbiome Bray–Curtis indices. The effects of both host environment (PERMANOVA, F(1) = 1.46, p = 0.001) and host genus (PERMANOVA, F(2) = 1.51, p = 0.012) were significant for metagenomic pool callitrichid gut microbiome beta diversity.

#### *L.rosalia* and *Callithrix* sp. metagenomic gut microbiome composition

**Figure 3:**
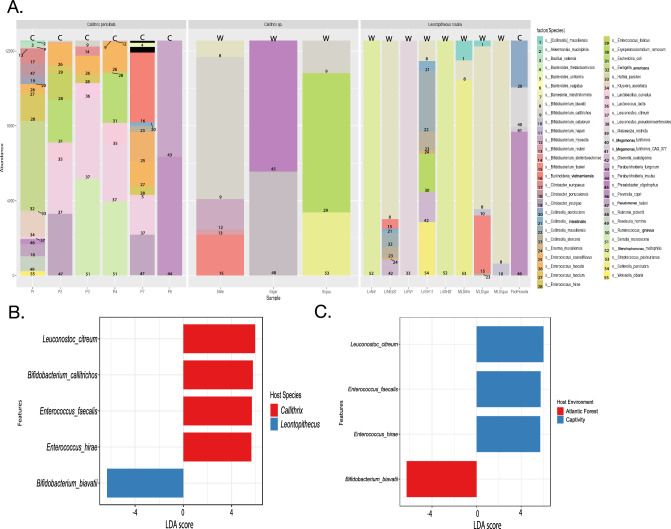
Gut metagenomic pools from callitrichids are enriched for *Bifidobacterium* species. (**A**) Relative levels of bacterial species abundance by host environment (W = Wild, C = Captive) and taxon classification of pooled samples. Each bacterial species represented in the legend on the right hand side is represented by a color and a number. Bacterial abundances for a specific bacterial species are labeled by the corresponding color and number in bars representing each individual gut metagenonic pool. Bacterial species with relatively ultra low abundances were blacked out in the bar representing gut metagenomic pool P7. (**B**). LefSe analysis of gut microbiome bacterial species abundance by host taxon of sampled pools. (**C**) LefSe analysis of gut microbiome bacterial species taxa abundance by host taxon of sampled pools.

The relative abundances of gut microbiome bacteria among callitrichid hosts are shown in Fig. 3A and absolute and relative abundance counts are also shown by host taxon in Supplementary Table S2 and by host environment in Supplementary Table S3. *Bifidobacterium* is more abundant in the gut microbiome of Atlantic Forest hosts than that of captive hosts. For example, *Bifidobacterium callitrichos* is more abundant in the former (see label “9” in bars for Safe and SIgua in Fig. 3A) than in the latter (see label “9” in bars for P1, P3, and P4 in Fig. 3A). Among GLT hosts, we see *Bifidobacterium biavatii* being the most abundant *Bifidobacterium* species in the gut of wild hosts, but *Bifidobacterium* is absent from the single captive gut microbiome GLT pool (Fig. 3A). *Bifidobacterium tissieri* is also in the gut microbiome of wild GLT hosts (abundance label “15” in LrNes2f and MLDAfe in Fig. 3A). For *Enterococcus*, the bacterial species most abundant in the gut of captive marmoset hosts were *Enterococcus faecalis*, *Enterococcus facium*, and *Enterococcus hirae* (see abundance labels “26–28” in bars representing P1–P4, and P7 in Fig. 3A). Only a single species of *Enterococcus*, *Enterococcus italicus*, was identified in a single gut microbiome pool of wild *Callithrix* hosts (bar for SIgua Fig. 3A). Then, *Serratia marcescens* was unique to gut microbiome pools of captive *Callithrix* hosts (see abundance label “51” in P3 and P4 bars in Fig. 3A), and *Megamonas funiformis* occurred only in the single captive GLT gut microbiome pool (see abundance labels “40–31” for PoolRosalia bar in Fig. 3A).

Results of LefSe differential abundance testing of callitrichid gut microbiome metagenomic pools show that gut microbiome pools from marmosets were enriched for *B. callitrichos* and three other bacterial species (Fig. 3B). GLTs were enriched for *B. biavatii* (Fig. 3B). Then LefSe analysis indicated that enriched gut microbiome bacterial species included *Enterococcus hirae* and *Enterococcus faecalis*, and *Leuconostoc citreium* among captive pool samples, and *B. biavatii* was among the enriched bacterial species in Atlantic Forest sample pools (Fig. 3C).

#### Gut microbiome functional pathways of *Callithrix* and *Leontopithecus* metagenomic pools

**Figure 4:**
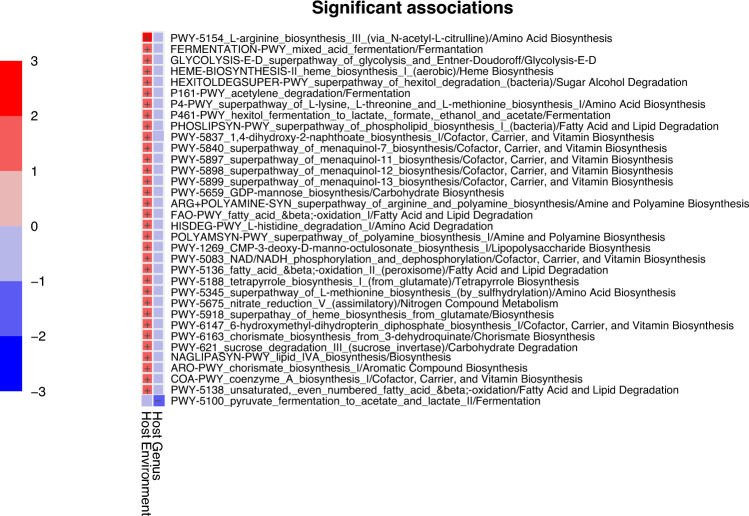
Callitrichid microbiome functional pathways are enriched for metabolite biosynthesis, degradation, and fermentation. Heatmap of MaAsLin analysis of significant associations Metacyc functional pathway superclasses and host environment and genus classifications of callitrichid sample pools, respectively. Level of association between each functional pathway and host category is indicated by the legend on the left side.

From the 1700+ metabolic pathways found in our data set, fitting a MaAsLin fixed effects model identified 38 Metacyc functional pathways with significant differential abundance among our gut metagenomic sample pools. We removed a total of five Metacyc pathways which were either attributed to non-bacterial organisms like plants or mammals or not identifiable within the Metacyc database. A heatmap of the finalized set of enriched pathways is shown in Fig. 4, along with the higher order functional Metacyc ‘superclass’ of each pathway. Enrichment information for each pathway within a specific host classification is given in Supplementary Table S4. With one exception, all these pathways were significantly enriched in captive callitrichids (Fig. 4). These pathways fell under Metacyc superclasses related to metabolite biosynthesis, degradation, and fermentation. More specifically, pathways included Entner–Doudoroff Glycolysis pathway and carbohydrate degradation. A single pathway was enriched in GLTs (PWY-5100) which was related to pyruvate fermentation.

**Figure 5:**
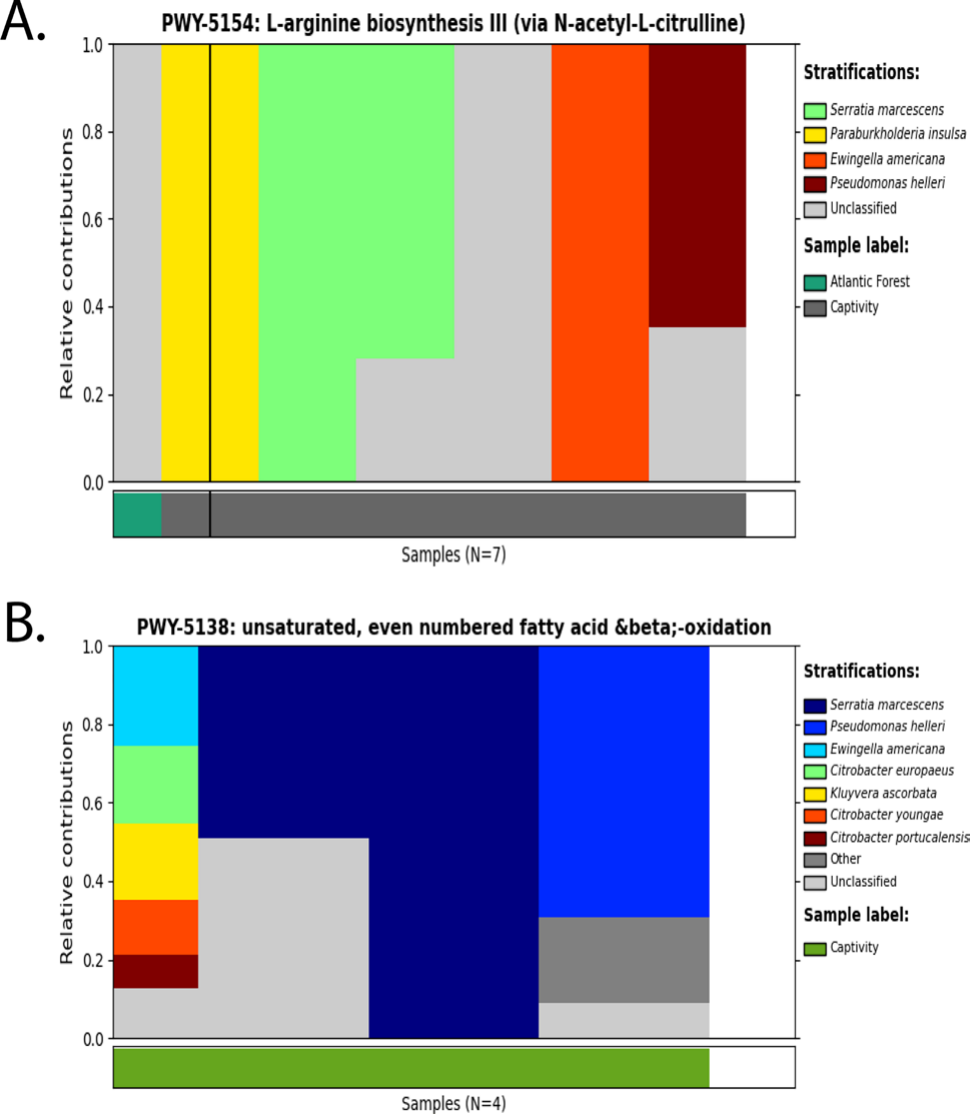
Relative abundance plots of gut microbiome bacterial contributions to differentially abundant functional Metacyc pathways. Each separate plot within the figure represents an individual pathway, each bar represents an individual sample pool, and stratification within each bar represent bacterial species.

Bacterial species associations with callitrichid gut microbiome functional pathways are shown in Fig. 5 and Supplementary Fig. S3. We see multiple associations of the same recurrent set of gut bacterial species across a wide range of Metacyc pathways. These bacterial species include *Serratia marcescens*, *Escherichia coli*, *Ewingella americana*, and *Pseudomonas helleri*, with most pathways being enriched for captivity. One exception is shown for Pathway PWY-5100, which is associated with pyruvate fermentation to acetate and lactate II (Supplementary Fig. S3). This pathway shows that *B. callitrichos* carries out this function in wild *Callithrix* host.

### MAG recovery from callitrichid metagenomic pools

MetaSPades (MEGAHIT results are shown in Supplementary Figs. S1 and S2) generated the following contig results from callitrichid gut metagenomic pools: (1) 637 co-assembled contigs (65,079 bp for the largest contig) with N50 of 2932 bp (base pairs) and L50 of 183 bp from wild GLTs; (2) 72 co-assembled contigs (92,239 bp for the largest contig) with N50 of 12,354 bp and L50 of 10 from wild marmosets; (3) 4146 co-assembled contigs (79,182 bp for the largest contig) with N50 of 4421 bp and L50 of 1008 from captive marmosets; and (4) 11 contigs (6058 for the largest contig) with N50 of 2949 bp and L50 of 4 from captive GLTs. MAXBIN2 produced 4 bins from captive marmoset metagenomic pools and 2 bins from wild GLT metagenomic pools, but failed to produce bins for the remaining data. CONCOCT produced 37 bins from captive marmoset metagenomic pools, 26 bins from wild GLT metagenomic pools, 8 bins from wild marmoset metagenomic pools, and 4 bins from the captive GLT metagenomic pool. The DAS Tool produced a total of 4 captive marmoset bins, but failed to produce optimized bins for captive GLTs, wild GLTs, and wild marmosets. Marker lineage beyond “root” during CheckM bin quality checks were only determined for the the 4 captive *Callithrix* optimized co-assembly bins (detailed information on these bins is available in Supplementary Table S5). Further MAG taxonomic and functional classification were only carried out for these 4 bins.

#### Taxonomic classification of MAGs from callitrichid metagenomic pools

Taxonomic classification of bins from captive marmoset hosts (Supplementary Table S5) placed two bins within the Gammaproteobacteria bacterial class, 1 bin in the the Actinomycetales bacterial class, and 1 bin into the Actinomycetia. All bins were classified down to the genus level (*Microbacterium*, *Serratia*, *Pseudomonas*, and *Leuconostoc*). One bin was classified at the species level as *Serratia marcescens*, which was the bin with the highest level of completeness and lowest level of contamination among these 4 bins (Supplementary Table S5).

#### Functional classification of MAGs from callitrichid metagenomic pools

**Figure 6:**
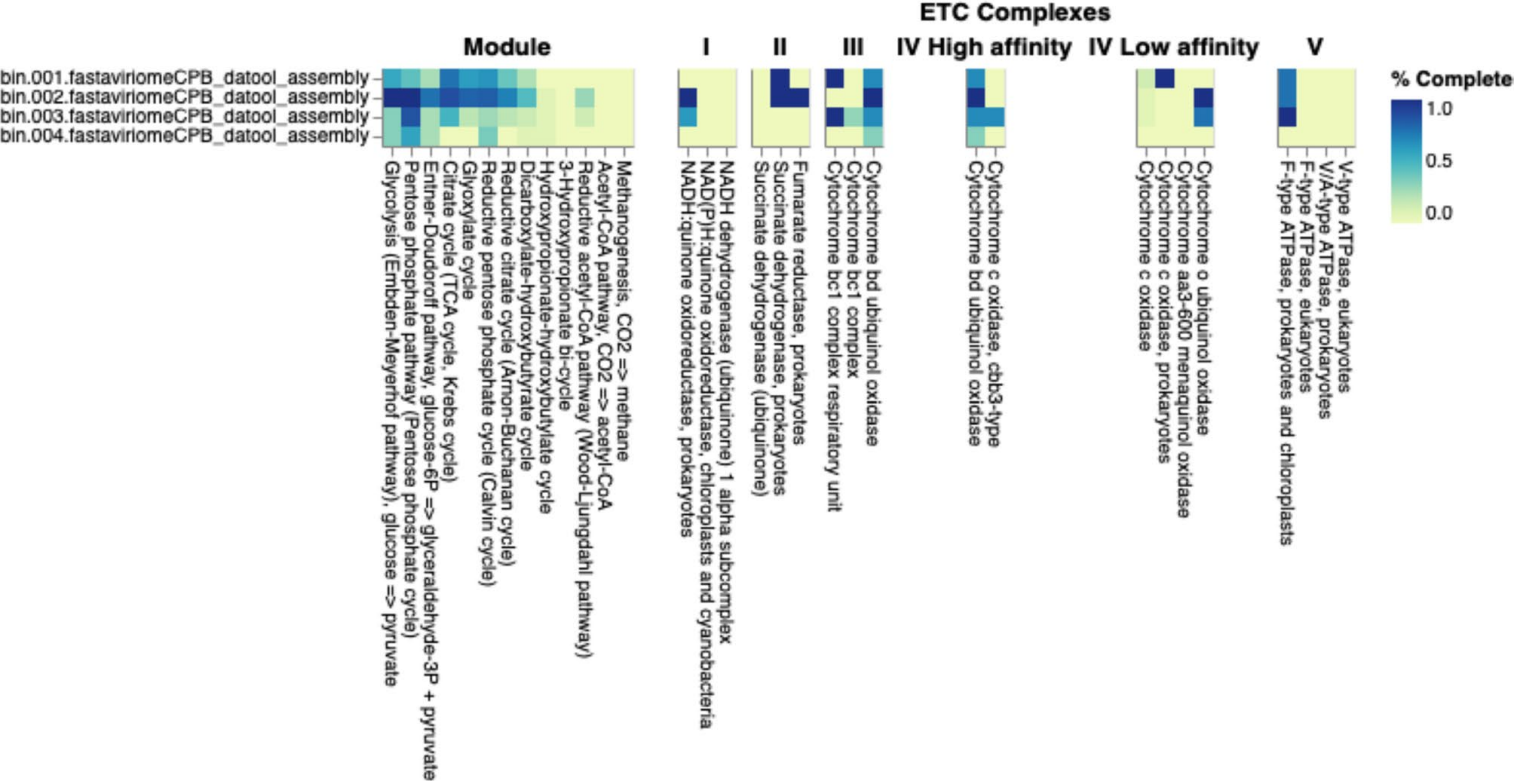
DRAM annotations of MAGs (metagenome-assembled genomes) recovered from four bins (bin.001–bin.004) of captive *Callithrix* gut metagenomic pools. The colors in the heatmap represent the completeness of relevant pathways and electron transport chain complexes (ETC) in each MAG. The heatmap was as part of DRAM output and shows modules present in at least one MAG.

**Figure 7:**
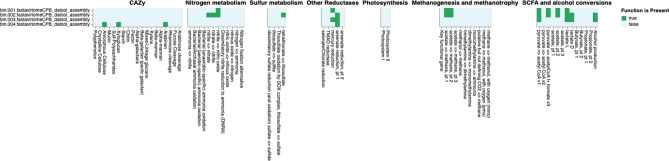
Presence/absence of relevant metabolic functions based on DRAM annotations of MAGs (metagenome-assembled genomes) recovered from four bins (bin.001–bin.004) of captive *Callithrix* gut metagenomic pools. The colours in the heatmap represent the presence or absence of relevant metabolic functions in each MAG. The heatmap was a part of DRAM output and shows modules present in at least one MAG. Abbreviations used in the figure are as follows: *CAZy* carbohydrate-active enzymes, *metab.* metabolism, *red.* reductases, *SCFA* short-chain fatty acids.

Based on DRAM annotations, genes related to glycolysis (Embden–Meyerhof pathway), pentose phosphate pathway (pentose phosphate cycle), citrate (TCA or Krebs cycle), glyoxylate, reductive pentose phosphate (Calvin cycle), reductive citrate (Arnon–Buchanan cycle), dicarboxylate–hydroxybutyratecycles, and the reductive acetyl-CoA pathway (Wood–Ljungdahl pathway) were found in the bin classified as *Serratia marcescens* (Fig. 6). The same set of pathways except the Wood–Ljungdahl pathway were also represented, but to a lesser degree of completeness, in the bins classified as *Microbacterium * and *Pseudomonas* (Fig. 6). Carbohydrate-active enzymes (CAZymes) genes were mostly absent from bins except for the bin classified as *Leuconostoc*, for which CAZymes for amorphous cellulose, xyloglucan, and arabinan were present (Fig. 7). *Serratia*, *Microbacterium*, and *Pseudomonas* bins possessed genes involved in several short chain fatty acids (SCFAs) and alcohol conversion modules. SCFA modules in these bins included those for pyruvate to acetyl CoAv1 conversion and lactate L. Supplementary Table S6 shows gene annotations for the four bins that represent recovered captive *Callithrix* gut microbiome MAGs. Supplementary Table S7 gives gene counts of functional modules across a wide variety of metabolisms based on DRAM annotations.

## Discussion

The aim of this study was to expand our compositional and functional knowledge of the callitrichid gut microbiome. Thereby, we analyzed the bacterial component of VLP-filtered metagenomic pools that were opportunistically available from GLTs and marmosets. Although such data are not conventionally utilized for microbiome studies, our utilization of these data was largely motivated by (1) the importance that a healthy gut microbiome may play in the conservation of endangered animals; (2) the threatened conservation status of GLTs; (3) the potential health impact of hybrid marmosets introduced to the GLT geographic distribution; and (4) the relatively small number of studies conducted on Brazilian primate gut microbiomes. Metatranscriptomic data represent transcribed bacteria, and seem to be dominated by a small set of bacterial genera^60^. VLP-filtered metagenomic pools, which are specifically processed to enrich for VLPs and ideally remove all other biological material present in a sample, are even likely more biased in their representation of a few select bacterial taxa. These limitations are important to keep in mind while interpreting the results obtained in this present study.

Despite these limitations and when specifically considering results from community profiling approaches, we observed a number of parallel patterns between our findings and previously available callitrichid metagenomic studies. First, we observe significant effects of host taxon on callitrichid gut microbiome alpha and beta diversity, which may be related to relative differences for exudivory specialization between callitrichid taxa^26,27,29,30^. A similar pattern was observed for gut microbiome diversity across and within several *Callithrix* species and hybrids^19^, which differ in their level of specialization for exudivory. In wild lemur species, alpha diversity of gut microbiome composition also was significantly influenced by host taxon^61^. Further microbiomes, metagenomes, and metabolomes have been found to be species-specific in lemurs and attuned to host dietary specializations and associated gastrointestinal morphology^61^. Significant differences in GLT and marmoset gut microbiome diversity are also likely related to differences in dietary specialization between hosts. For callitrichids, a similar systematic study of taxa adapted to different dietary strategies as well as differing levels of exudivory specialization is necessary. Undertaking such work, especially in wild populations, will allow us to better understand how host phylogeny influences gut microbiome diversity.

In general, gut microbiome studies show that there is a significant difference in gut microbiome diversity between captive and wild hosts (marmosets^19^, kiwis^62^, Tasmanian devil^63^, mice^64^, primates^22^, raptors^65^, rhinos^66^, woodrats^67^). For our callitrichid data set, we found a significant effect of host environment on gut microbiome beta diversity but not alpha diversity. Cluster analysis of our data in fact shows almost no overlap of beta-diversity measures between captive and wild hosts, independent of host taxon. We do note that the effect of this host variable on gut alpha diversity was nearly significant, and it is likely that a larger sample size for our data would have produced a significant effect of host environment on callitrichid gut microbiome alpha diversity. Several previous studies agree that dietary differences between host captive and wild environments are one of the main factors driving some of these gut microbiome changes^22,62–70^.

In terms of bacterial taxa abundance in our metagenomic pool data set, we hypothesized that GLT and *Callithrix* gut microbiomes are enriched for *Bifidobacterium*, a bacterial genus important for host carbohydrate metabolism^21,71,72^. Our hypothesis is based on evidence that Neotropical primates gut microbiomes are significantly enriched for this bacterial genus relative to Old World primates^73^. Further *Callithrix* and *Leontopithecus* are the two primate genera with the highest average abundance of *Bifidobacterium* in the primate gut microbiome^21^. Based on community profiling, we found gut microbiome metagenomic pools of wild GLTs and *Callithrix* sp. to be significantly enriched for *Bifidobacterium* relative to their captive counterparts, a pattern which has been previously observed in the gut microbiome of wild and captive *Callithrix*^19^. Several other captive *C. jacchus* studies have also shown that *Bifidobacterium* plays an important compositional and functional role, particularly for carbohydrate mechanism, in *Callithrix*^21,74^. The results from our study extend this observation to also include the gut microbiome of GLTs. As our results provide further evidence of the importance of *Bifidobacterium* in the callitrichid gut, our current work and several other studies support to the idea that an important evolutionary relationship exists between the Callitrichidae family and *Bifidobacterium*^21^.

The types of *Bifidobacterium * species of that inhabit the gut microbiome of Neotropical primates seem to vary and depend on the taxon of the host^73^. While one recent study of the gut microbiome of wild and captive marmosets^19^ could only determine that bacteria from the *Bifidobacterium* genus were present within the gut microbiome of sampled hosts, captive studies have found that *B. callitrichos* and *B. myosotis* are common within the *Callithrix* gut microbiome^21,72–74^. We also show that *Callithrix* gut microbiome metagenomic pools were specifically enriched for *B. callitrichos* and *B. myosotis*. Strains of *B. callitrichos* previously found in the gut of captive *C. jacchus* were thought to possess genes that contributed to galactose, arabinose, and trehalose metabolic pathways^74^. Further, different *B. callitrichos* strains with significant genomic differences were found within the same marmoset host, results that suggest that different *Bifidobacterium* strains support various roles for carbohydrate metabolism within individual hosts^74^. *Bifidobacterium myosotis* is a relatively recently recognized species of *Bifidobacterium* that was identified in the feces of a baby *C. jacchus*^72^. Our data also indicated that gut metagenomic pools from Atlantic Forest hosts and GLTs were enriched for *B. biavatii*. This bacterial species has been isolated from the red-handed tamarin *Saguinus midas*, but interestingly, *B. biavatii* is common among primate taxa in general^21^.

By applying metagenomic community profiling approaches, we observed significant enrichment and high relative abundance of other bacterial species besides *Bifidobacterium* in callitrichid gut metagenomic pools. Metagenomic pools from hosts that were both *Callithrix* and captive were significantly enriched for *Leuconostoc citreum*, *Enterococcus facelis*, and *Enterococcus hirae*. *Leuconostoc citreum* is considered a lactic acid bacterium, a bacterial type known to be involved in sugar fermentation and that can colonize the gut^75^. Some lactic acid bacteria are able to metabolize oligosaccharides^76,77^. *Leuconostoc citreum* produces SCFAs^78^, a function also observed in *Bifidobacterium*, which may guard against the proliferation of pathogenic bacteria in the gut and decrease chronic inflammation^79,80^. It is plausible that within our sample of captive *Callithrix*, lactic acid bacteria may provide some of the same functional and protective properties in the gut as provided by *Bifidobacterium * to the gut of wild marmoset hosts. *Enterococcus* is present in the gastrointestinal tract of human and non-human animals, usually serving as commensals that participate in metabolism of carbohydrates and other nutrients but can turn into opportunistic pathogens in other environments^81^. *Enterococcus facelis* has been found in the enclosures of captive *C. jacchus*, which was recognized as a potential but not active ‘veterinary risk’^82^. *Serratia marcescens*, which was found in two captive *Callithrix* gut metagenomic pools, has been found to be injurious to intestinal epithelial cells in humans^83^. Finally, the only host category to possess *Megamonas funiformis*, another potentially pathogenic bacterial species, was found in the captive GLT gut microbiome pool. Other gut microbial community studies have associated *Megamonas* with obesity, inflammation, and prediabetes (see the discussion in^84^).

Relative to our community profiling approach, co-assembly MAG recovery approaches provided much sparser results for callitrichid gut metagenomic pools. For example, we were only able to classify bacterial species for co-assembled bins recovered from gut metagenomic pools of captive *Callithrix* hosts. Given that our data set was initially enriched for VLPs, the resulting filtered data most likely do not possesses a sufficient number of sequencing reads from the bacterial component to produce high quality MAGs. This point is evident from the low number of co-assembled contigs obtained from the majority of callitrichid gut metagenomic pools. However, some of the bacterial taxa classified through MAG recovery in bins from the gut of captive marmoset hosts (*Enterococcus*, *Pseudomonas*, and *Serratia marcescens*) overlapped with taxa also identified by community profiling approaches. However, except for the *Serratia marcescens* bin, all other bins did not meet MIMAG standards of completeness (> 90%) and contamination (< 5%) for high-quality MAG drafts^52^.

Functional analysis of callitrichid gut microbiome metagenomic pools via community profiling showed enrichment of 38 Metacyc pathways related to biosynthesis, degradation, and fermentation of metabolites. It is important to keep in mind that our functional results only give a partial look into the microbiome of sampled GLTs and marmosets. Functional pathways related to carbohydrate metabolism included those for pyruvate fermentation, carbohydrate degradation, and sugar alcohol degradation. In terms of host environment, functional pathways were enriched in captive but not wild callitrichids. For host genus, a single functional pathways was enriched in GLTs. Given that our data set only represents a select group of bacteria from callitrichid guts, these results likely show a limited representation of functional pathway enrichment of different classes of callitrichid hosts. However, we do see some parallel patterns in results with that of Malukiewicz et al.^19^ as to which bacterial species are preforming which functional roles within the gut of callitrichids. The latter study showed that whereas *Bifidobacterium* plays an important role in carbohydrate metabolism in wild *Callithrix*, other bacterial species carry out these functions in captive *Callithrix*. Our data indicate that pathways related to carbohydrate metabolism as well as other functions is carried out in captive callitrichids by *Serratia marcescens*, *Escherichia coli* *Pseudomonas helleri*, and *Ewingella americana*.

Despite the lack of high quality MAG recovery for our data set, functional annotation was possible for some of the recovered MAGs. For example, functional annotation of genes in the * Serratia marcescens* bin pointed to a role of the bacterial species in pyruvate fermentation in captive *Callithrix* hosts. Interestingly, functional annotation via community profiling pointed to pyruvate fermentation in the gut microbiome being carried out by *Bifidobacterium callithricos* in a metagenomic pool from one of the wild *Callithrix* hosts. These results point to the likelihood that metabolic functions usually carried out by *Bifidobacterium* in the gut of wild marmoset hosts are carried out by potentially pathogenic bacteria in the gut of captive marmoset hots.

The major findings of this study are consistent with previous studies in showing that *Bifidobacterium* is an important component of the callitrichid gut microbiome, and that the composition of GLT and marmoset gut microbiota is sensitive to host environmental factors. It will be, nonetheless, important for future studies to further confirm, replicate, as well as build upon our findings due to some of the inherent limitations of our opportunistic data set. A major goal of integrating the study of microbiomes into conservation research is determining what indicates a “healthy baseline” microbiome for a given host taxon^9^. This task requires the determination of reliable microbial indicators that consider the specific conservation needs of the host^9^. Given that urbanization and land use are considered major threats to GLT conservation, we recommend that future studies focus on the composition and functional aspects of the GLT gut microbiome among forest fragments which vary in terms of factors such as size, level of degradation, dietary intake, access to nutritional resources and proximity to urbanized areas. Ultimately, such data should facilitate the not so simple tasks of distinguishing between the causes and effects of community changes, and determination of whether these changes are functionally consequential for the host^9^.

For gut microbiome studies of marmosets, with the exception of this study and that of^19^, other studies are highly biased towards *C. jacchus*, such that future studies should strive to expand sampling to other marmoset species. Hybridization is also an extremely common occurrence in marmosets, which should be further explored in relation to the marmoset microbiome. This data set was highly biased toward *C.* *jacchus* x* C. penicillata* hybrids, expanding sampling of other types of free-ranging and captive marmoset hybrids is necessary to move marmoset microbiome studies forward. Overall, such information will expand baseline gut microbiome data available for wild and non-wild callitrichids to allow for the development of new tools to improve their management, welfare, and conservation. For new research into the callitrichid microbiome, we especially recommend utilizing shotgun whole metagenomic and/or transcriptomic approaches (which naturally do not intentionally filter out bacteria) in lieu of 16s rRNA approaches that have been previously utilized to significantly increase the inferential power to characterized the functional as well as taxonomic aspects of the callitrichid gut microbiome^85,86^.

### Supplementary Information

Supplementary Table S1.

Supplementary Table S2.

Supplementary Table S3.

Supplementary Table S4.

Supplementary Table S5.

Supplementary Table S6.

Supplementary Table S7.

Supplementary Figure S1.

Supplementary Figure S2.

Supplementary Figure S3.

Supplementary Legends.

## Data Availability

Supplementary material for this work is available at https://doi.org/10.5281/zenodo.8271171. The data set supporting the conclusions of this article is available in the NCBI SRA repository under Bioproject PRJNA847605 (SAMN28946310–SAMN28946330). The authors declare that they have no competing interests. Supplementary material for this work is available at https://doi.org/10.5281/zenodo.8271171.
