# Supplementary material for: Bifidobacteria define gut microbiome profiles of golden lion tamarin (Leontopithecus rosalia) and marmoset (Callithrix sp.) metagenomic shotgun pools
Source: Sci Rep. 2023 Sep 21;13:15679. doi: 10.1038/s41598-023-42059-4 (PMC10514281; doi:10.1038/s41598-023-42059-4)
Supplement: Supplementary file 8 — Supplementary Figure S1. [file 41598_2023_42059_MOESM8_ESM.pdf]

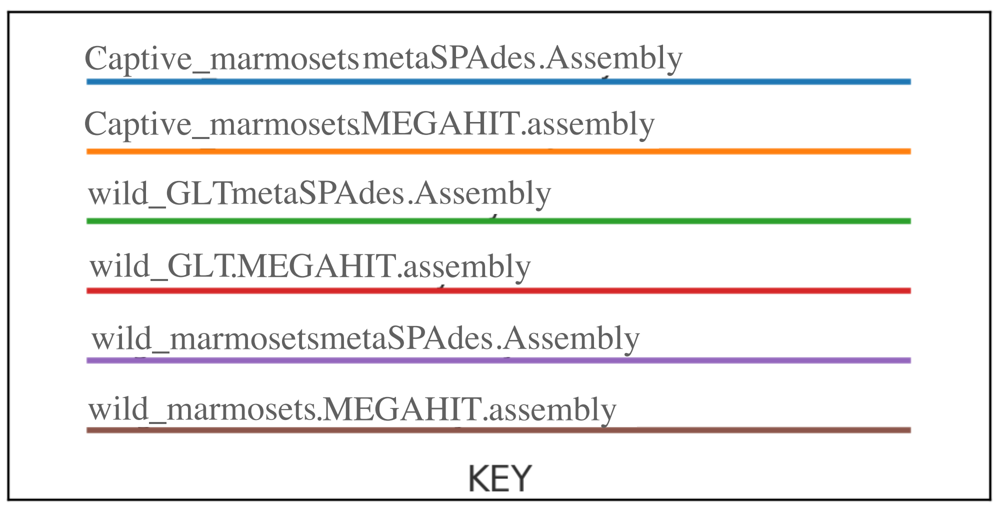

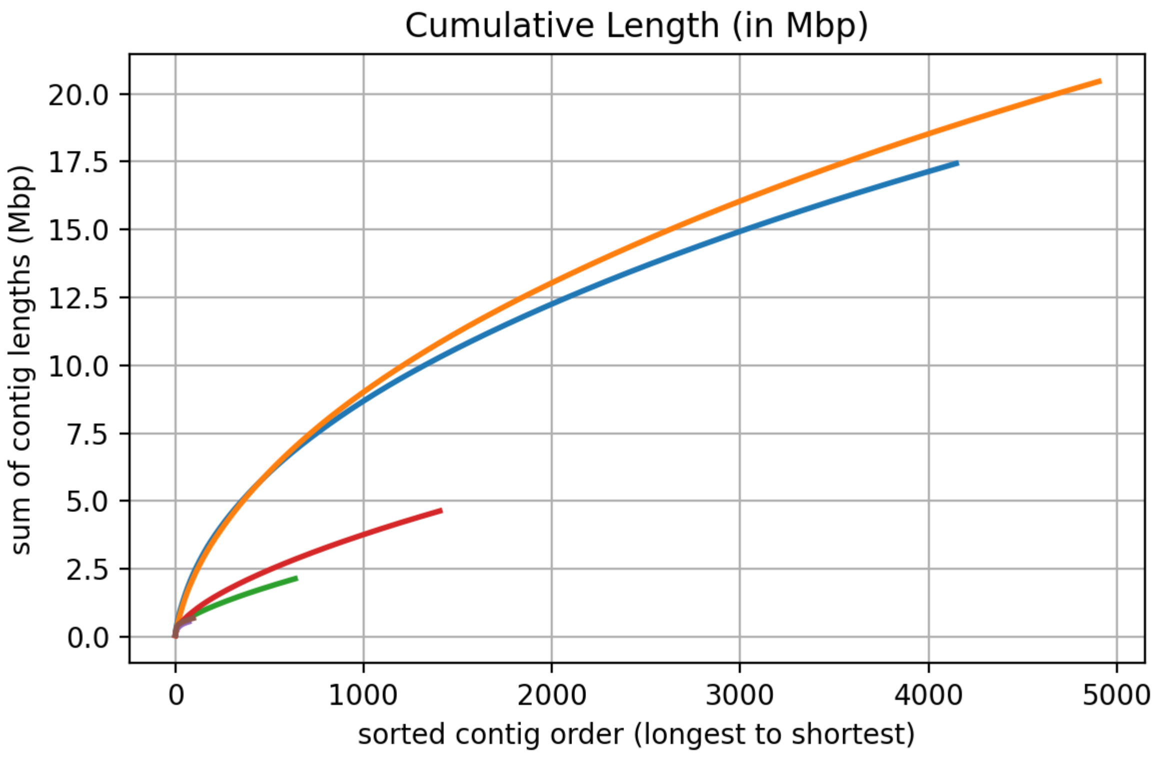

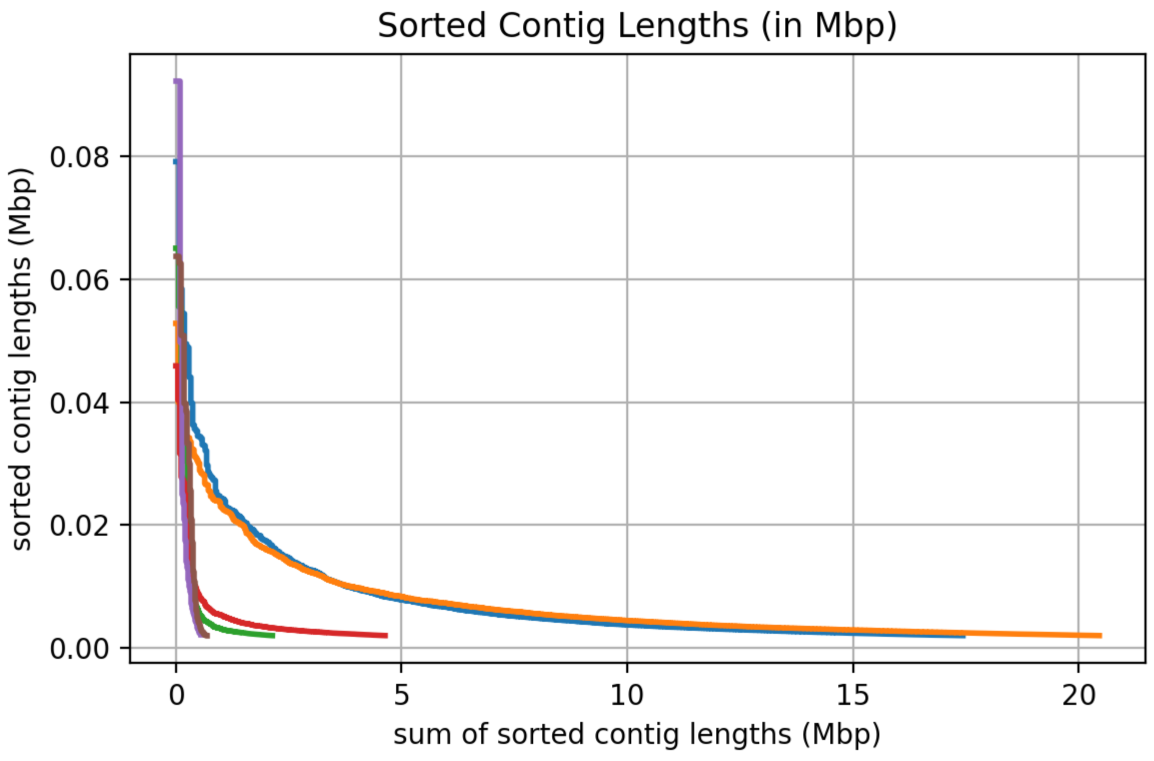

BEST WORST

ASSEMBLY	LONGEST CONTIG (bp)	Nx (Lx)		LENGTH (bp)	NUM CONTIGS	SUM LENGTH (bp)	Contig Length Histogram (1bp <= len < 10Kbp)	Contig Length Histogram (10Kbp <= len < 100Kbp)	Contig Length Histogram (len >= 100Kbp)
Captive_marmosets.metaSPAdes.Assembly	79182	N50:	4421	>= 10^6	0	0	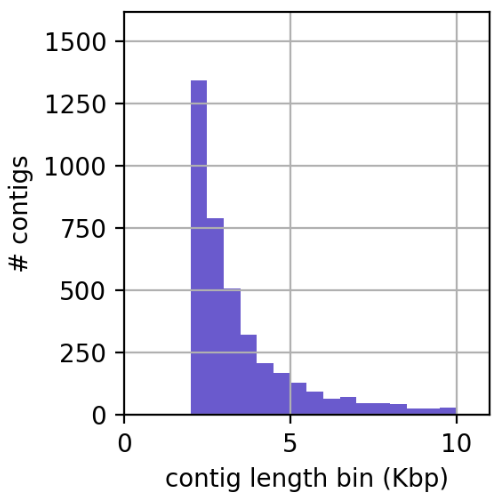	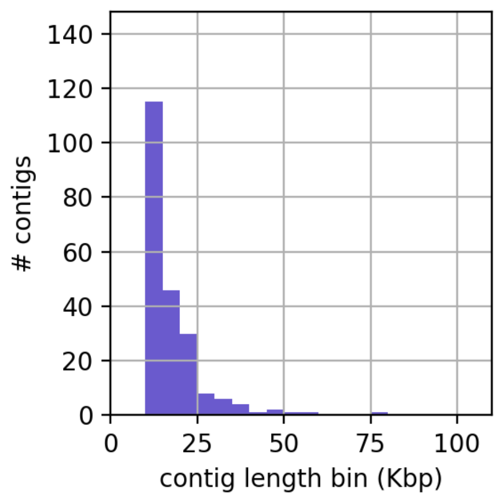	
		L50:	(1008)	>= 10^5	0	0			
		N75:	2805	>= 10^4	215	3782511			
		L75:	(2282)	>= 10^3	4146	17435014			
		N90:	2263	>= 500	4146	17435014			
		L90:	(3325)	>= 1	4146	17435014			
Captive_marmosets.MEGAHIT.assembly	52839	N50:	4374	>= 10^6	0	0	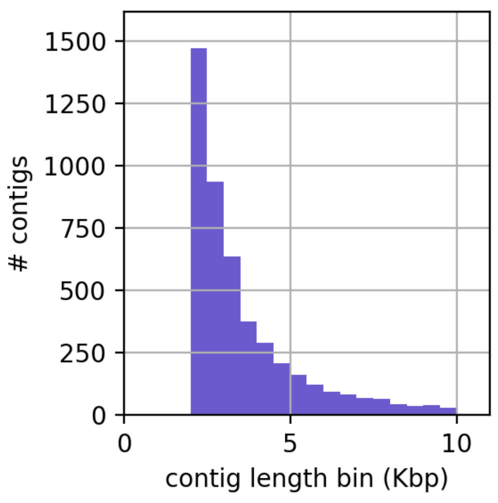	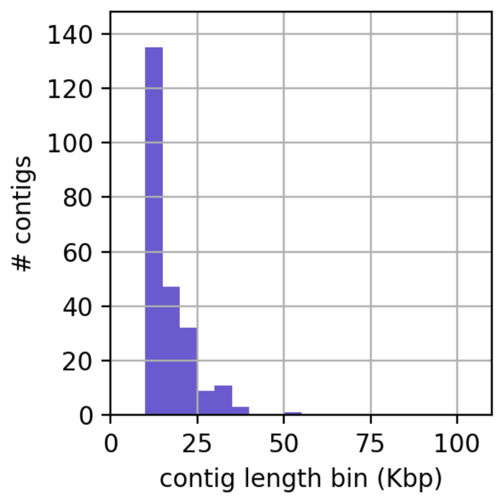	
		L50:	(1262)	>= 10^5	0	0			
		N75:	2856	>= 10^4	238	3888728			
		L75:	(2745)	>= 10^3	4903	20455038			
		N90:	2293	>= 500	4903	20455038			
		L90:	(3948)	>= 1	4903	20455038			
wild_GLT.metaSPAdes.Assembly	65079	N50:	2932	>= 10^6	0	0	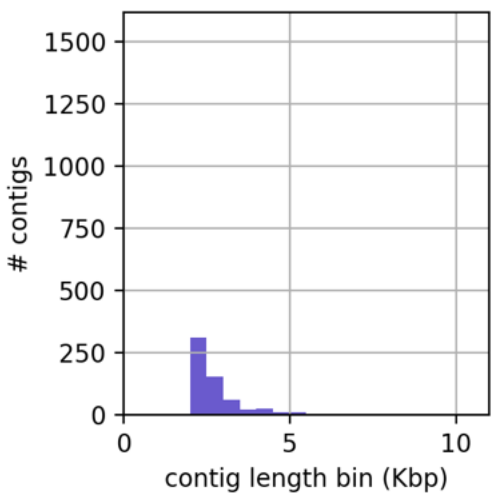	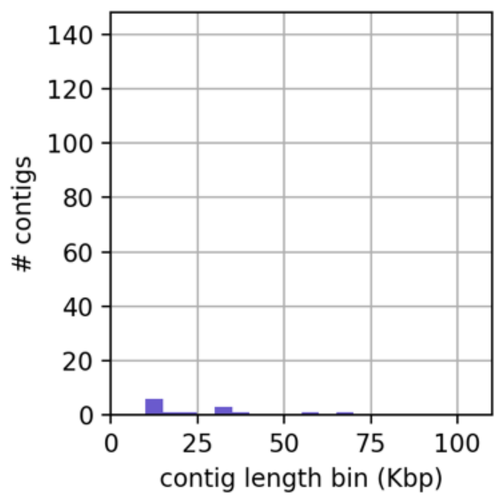	
		L50:	(183)	>= 10^5	0	0			
		N75:	2340	>= 10^4	14	356800			
		L75:	(389)	>= 10^3	637	2140135			
		N90:	2119	>= 500	637	2140135			
		L90:	(533)	>= 1	637	2140135			
wild_GLT.MEGAHIT.assembly	45927	N50:	3096	>= 10^6	0	0	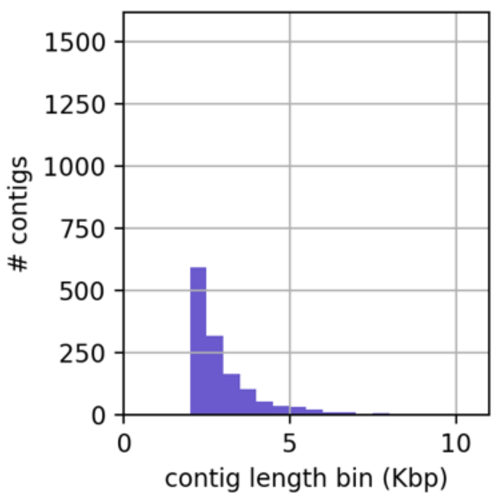	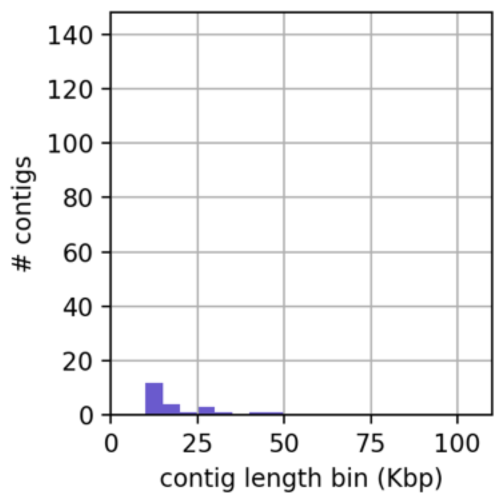	
		L50:	(451)	>= 10^5	0	0			
		N75:	2413	>= 10^4	23	436313			
		L75:	(878)	>= 10^3	1405	4635601			
		N90:	2159	>= 500	1405	4635601			
		L90:	(1182)	>= 1	1405	4635601			
wild_marmosets.metaSPAdes.Assembly	92239	N50:	12354	>= 10^6	0	0	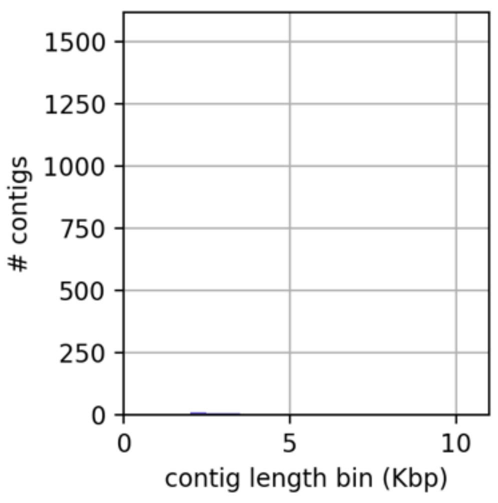	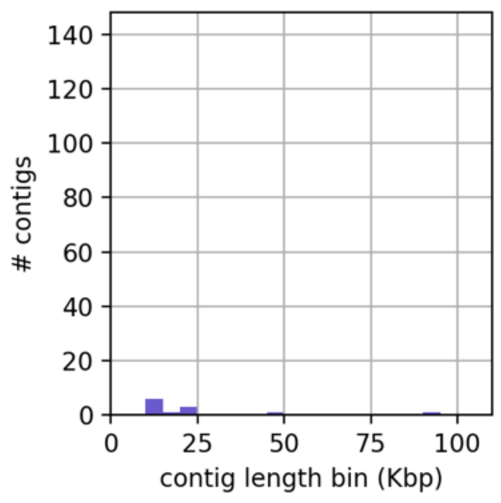	
		L50:	(10)	>= 10^5	0	0			
		N75:	5248	>= 10^4	12	303063			
		L75:	(29)	>= 10^3	72	555489			
		N90:	3018	>= 500	72	555489			
		L90:	(50)	>= 1	72	555489			
wild_marmosets.MEGAHIT.assembly	63796	N50:	25568	>= 10^6	0	0	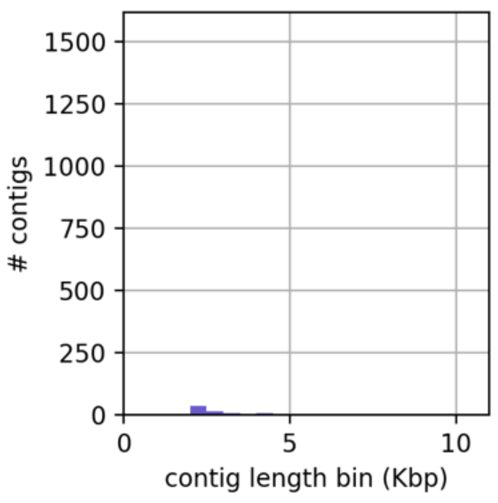	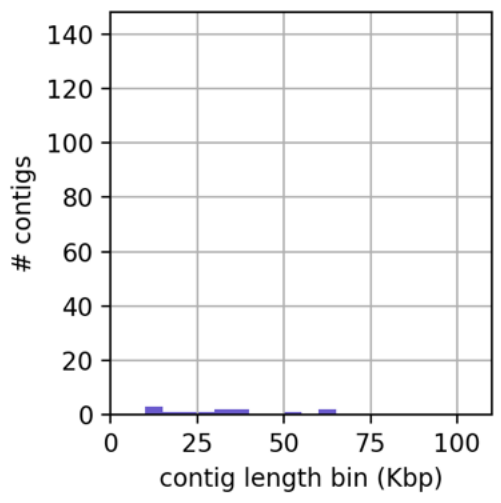	
		L50:	(8)	>= 10^5	0	0			
		N75:	4062	>= 10^4	13	416608			
		L75:	(31)	>= 10^3	96	685050			
		N90:	2344	>= 500	96	685050			
		L90:	(65)	>= 1	96	685050			
