# Supplementary material for: Bifidobacteria define gut microbiome profiles of golden lion tamarin (Leontopithecus rosalia) and marmoset (Callithrix sp.) metagenomic shotgun pools
Source: Sci Rep. 2023 Sep 21;13:15679. doi: 10.1038/s41598-023-42059-4 (PMC10514281; doi:10.1038/s41598-023-42059-4)
Supplement: Supplementary file 9 — Supplementary Figure S2. [file 41598_2023_42059_MOESM9_ESM.pdf]

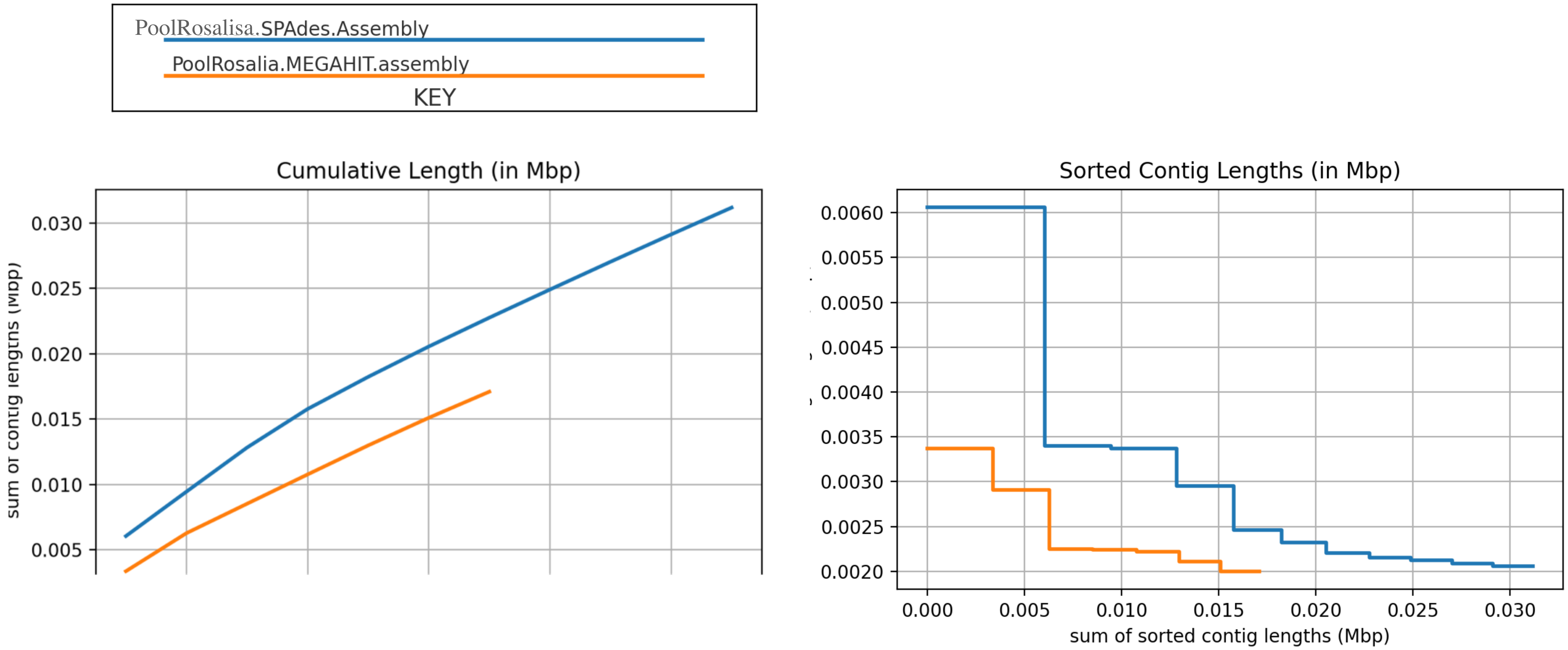

BEST | | | | | | | | WORST

ASSEMBLY	LONGEST CONTIG (bp)	Nx (Lx)		LENGTH (bp)	NUM CONTIGS	SUM LENGTH (bp)	Contig Length Histogram (1bp <= len < 10Kbp)	Contig Length Histogram (10Kbp <= len < 100Kbp)	Contig Length Histogram (len >= 100Kbp)
PoolRosalisa.metaSPAdes.Assembly	6058	N50:	2949	>= 10^6	0	0	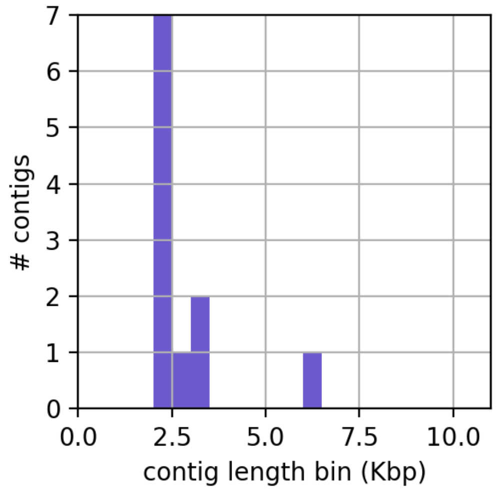		
		L50:	(4)	>= 10^5	0	0			
		N75:	2149	>= 10^4	0	0			
		L75:	(8)	>= 10^3	11	31180			
		N90:	2088	>= 500	11	31180			
		L90:	(10)	>= 1	11	31180			
PoolRosalia.MEGAHIT.assembly	3371	N50:	2241	>= 10^6	0	0	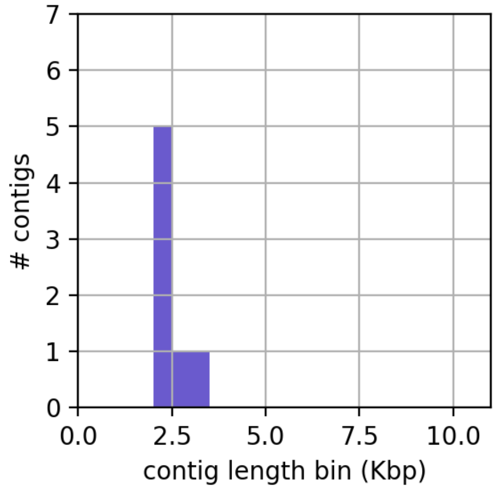		
		L50:	(4)	>= 10^5	0	0			
		N75:	2219	>= 10^4	0	0			
		L75:	(5)	>= 10^3	7	17102			
		N90:	2000	>= 500	7	17102			
		L90:	(7)	>= 1	7	17102			
