# Supplementary figures and images for: Bifidobacteria define gut microbiome profiles of golden lion tamarin (Leontopithecus rosalia) and marmoset (Callithrix sp.) metagenomic shotgun pools
Source: Sci Rep. 2023 Sep 21;13:15679. doi: 10.1038/s41598-023-42059-4 (PMC10514281; doi:10.1038/s41598-023-42059-4)

A.

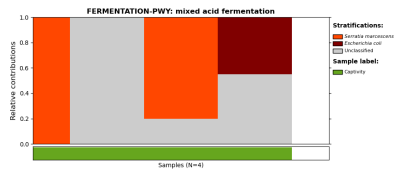

B.

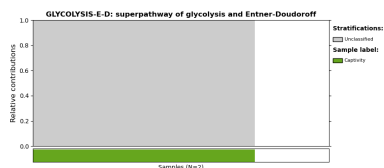

C.

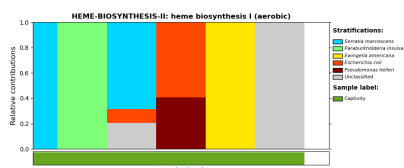

D.

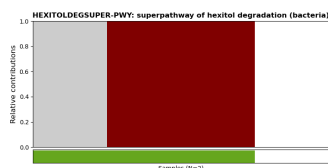

E.

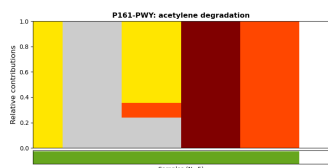

F.

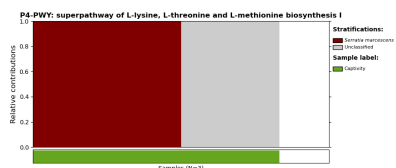

G.

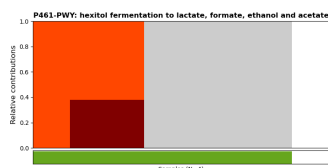

H.

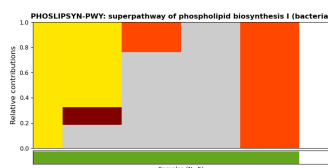

I.

J.

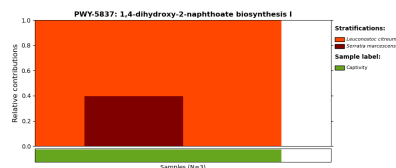

K.

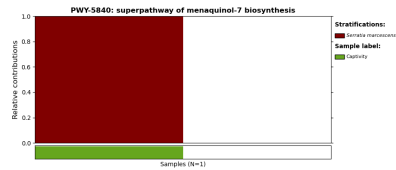

L.

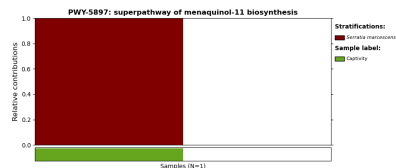

M.

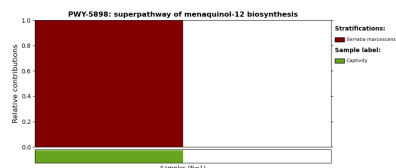

N.

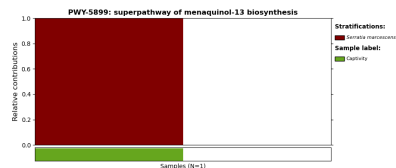

O.

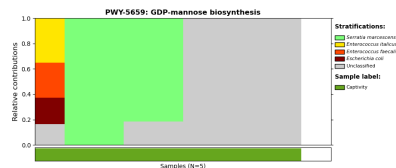

P.

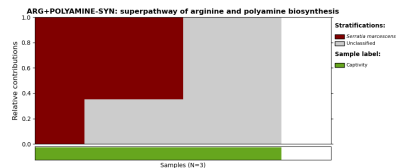

Q.

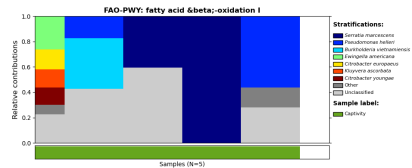

R.

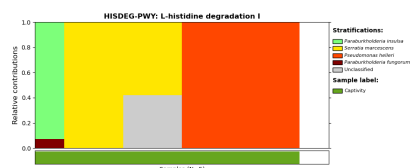

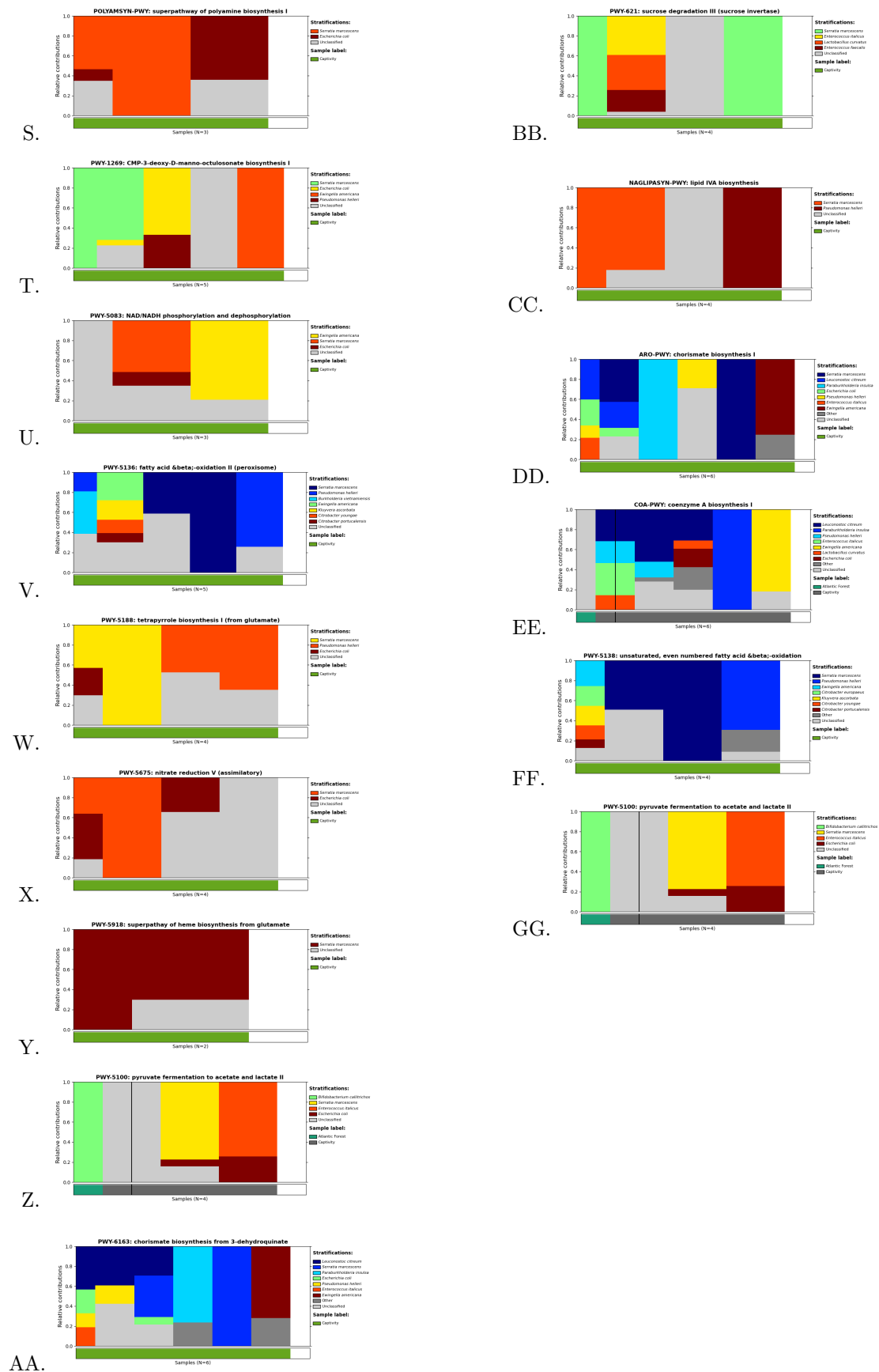

Supplement: Supplementary file 10 — Supplementary Figure S3. [file 41598_2023_42059_MOESM10_ESM.pdf]
